# Unveiling the strong positive relationship: Maternal characteristics and neonatal outcomes in the Better Outcomes in Labour Difficulty (BOLD) study – a secondary analysis validating neonatal near miss classification

**DOI:** 10.7189/jogh.14.04024

**Published:** 2024-01-19

**Authors:** Vicky Nogueira-Pileggi, Olufemi T Oladapo, João Paulo Souza, Hayala Cristina Cavenague de Souza, Cynthia Pileggi-Castro, Lawal O Oyeneyin, Livia Oliveira-Ciabati, Francisco Barbosa, José Simon Camelo

**Affiliations:** 1 Department of Paediatrics – Ribeirão Preto Medical School, University of São Paulo, São Paulo, Brazil; 2 UNDP, UNFPA, UNICEF, WHO, World Bank Special Programme of Research, Development and Research Training in Human Reproduction (HRP), Department of Reproductive Health and Research, Geneva, Switzerland; 3 Department of Social Medicine – Ribeirão Preto Medical School, University of São Paulo, São Paulo, Brazil; 4 Department of Obstetrics and Gynaecology, University of Medical Sciences Teaching Hospital, Ondo State, Nigeria

## Abstract

**Background:**

The near miss concept, denoting near collisions between aircraft, originated in aeronautics, but has recently been transferred to the neonatal context as a way of evaluating the quality of health services for newborns, especially in settings with reduced child mortality. However, there is yet no consensus regarding the underlying criteria. The most common indicators used to assess health care quality include mortality (maternal and neonatal) and life-threatening conditions. Using the World Health Organization (WHO) Better Outcomes in Labour Difficulty (BOLD) prospective cohort study data set, we conducted a secondary analysis to validate the near miss concept and explore the association between maternal and neonatal outcomes.

**Methods:**

We studied 10 203 singleton mothers treated between December 2014 and November 2015 in nine Nigerian and four Ugandan hospitals. We validated the near miss concept by testing the diagnostic accuracy (sensitivity, specificity, positive likelihood ratio, negative likelihood ratio, and odds ratio (OR)) using death as the reference variable and calculating the maternal and neonatal case fatality rates. We performed ordinal and binomial logistic regression, with the independent variables being those that had *P* < 0.1 in the univariate analyses. We considered the significance level of 5%.

**Results:**

We validated the neonatal near miss concept using the BOLD study data. We observed maternal and neonatal case fatality rates of 70.2% and 6.5%, with an increasing severity relationship between maternal and neonatal outcomes (*P* < 0.05). Ordinal logistic regression showed that gestational age <37 or >41 weeks and <8 antenatal consultations were related to a higher risk of neonatal severe outcomes, while maternal age between 30 and 34 years functioned as a protective factor against severe neonatal outcomes (SNO). Binomial logistic regression showed gestational age <37(OR = 1.46; 95% confidence interval (CI) = 1.07–1.94) or >41 weeks (OR = 2.26; 95% CI = 1.55–3.20), low educational level (OR = 1.76; 95% CI = 1.12–2.69), overweight/obesity (OR = 1.23; 95% CI = 1.02–1.47), one previous cesarean section (OR = 1.90; 95% CI = 1.36–2.61), one previous abortion (OR = 1.25; 95% CI = 1.00–1.56), and previous chronic condition (OR = 1.83; 95% CI = 1.37–2.41) were risk factors for SNO.

**Conclusions:**

The neonatal near miss concept could be used as a parameter for analysis in different health systems, to ensure that measuring of neonatal severity is comparable across health care units. In this analysis, we observed a progressive association between maternal severity and the severity of the newborns’ outcomes.

In 2016, the United Nations (UN) launched the Sustainable Development Goals (SDGs) for 2030 with the aim of fostering human development. In view of maternal health, SDG 3.1 focuses on reducing the global maternal mortality ratio to less than 70 per 100 000 live births, while SDG 3.2 seeks to end preventable deaths of newborns and children <5 years of age. To achieve this, countries should strive to reduce neonatal mortality to 12 per 1000 live births or less and under-five mortality to 25 per 1000 live births or less [1].

According to an UN report published in 2015, 16 000 children still die of preventable causes each year, with almost three out of four neonatal deaths caused by complications related to preterm births (35%) or labor and childbirth (24%) and sepsis (15%) [2]. Half of these deaths occur in Africa and Asia, whose population is expected to increase in the coming years.

Meanwhile, 86% of maternal deaths worldwide occur in sub-Saharan Africa and South Asia; most occur due to preventable reasons, such as postpartum hemorrhage, with inadequacies or shortages in antenatal care during pregnancy, skilled birth attending, and support in the weeks preceding birth acting as contributing factors [3].

Studies have demonstrated that, due to the high risk of death for both mothers and neonates, focusing on the day of birth could help improve their health [4]. At this point, the most commonly used indicators for assessing the quality of care are maternal and neonatal mortality and life-threatening conditions (e.g. severe maternal morbidity, near miss cases).

The near miss concept, used in aeronautics to denote near collisions between aircraft, was first mentioned in maternal health by Stones et al. [5] and has been in use since 2009. It has only recently been applied to the neonatal context concept, and no consensus is present in the field regarding its underlying criteria. Similar to its use in maternal health, the near miss concept allows for the evaluation of the quality of health services in neonatal care, especially in the settings where child mortality has already been reduced.

A systematic review conducted in 2015 sough to identify studies that used the neonatal near miss concept. Per one definition, it refers to a critical incident in the neonatal phase where an infant's survival was threatened, either through diseases, medical interventions, or organ dysfunctions. Another relates to a newborn who narrowly survived, but endured a serious birth complication either during delivery or within the initial seven days of life outside the womb. The review suggested the adoption of three key criteria identified in the largest World Health Organization (WHO) study – an Apgar score <7, birthweight <1750 g, and gestational age <33 weeks – as they are part of routine vital health indicators that can be retrospectively assessed [6].

The WHO Better Outcomes in Labour Difficulty (BOLD) study, conceptualised in 2014, aimed to reduce the number of maternal and neonatal deaths and morbidities, addressing critical barriers to good quality intrapartum care and strengthening the connection between health systems and communities [7-9]. With the development of the SDGs, BOLD highlighted the importance of maternal and child health, providing lessons for improvements in understanding and addressing the health indicators of maternal and neonatal population.

By performing a secondary analysis of BOLD data, we aim to validate the neonatal near miss concept and to explore the association between maternal and neonatal outcomes [9].

## METHODS

We used the data set produced within the BOLD project, which collected data in 13 hospitals in Africa (nine in Nigeria and four in Uganda) over 11 months (December 2014 to November 2015). The facilities were chosen based on the number of births and on the care provided (e.g. professional capacity, having access to a cesarean-section, good practices at birth among others). The study included women with singleton pregnancy admitted at first stage of spontaneous labor with a cervical dilatation <7 cm who had previously provided informed consent. It excluded mothers who previously had fetal death, were at advanced first stage of labor, had multiple pregnancies, gestational age less than 34 weeks, elective or pre-labor cesarean section, as well as minors without a guardian or who were not emancipated and those incapable of giving consent. The data were managed using RedCap [10]. Details about the population and the data collection tool are published elsewhere [7].

We used the following definitions and terminology:

− Maternal near miss (MNM): Refers to a woman who nearly died but survived a complication that occurred during pregnancy, childbirth or within 42 days of pregnancy termination;
− Maternal death: Death of a woman while pregnant or within 42 days of pregnancy termination or its management, but not from accidental or incidental causes;
− Severe maternal outcome: A life-threatening condition (i.e. organ dysfunction), including all maternal deaths and maternal near miss cases [11];
− Neonatal near miss (NNM): Refers to a newborn that nearly died but survived a complication that occurred during childbirth or within the week after birth, as proposed by Pileggi-Castro et al. [12].

We defined neonatal near miss criteria as a newborn having at least one of the following:

− Apgar score of <7 at five minutes;
− Birth weight of <1750 g;
− Gestational age of <33 weeks (pragmatic criteria);
− The use of antibiotics, anticonvulsants, or corticosteroids;
− Any intubation;
− Use of continuous positive airway pressure (CPAP);
− Phototherapy in the first 24 hours of life;
− A need for cardiopulmonary resuscitation (CPR);
− Use of surfactant;
− Usage of blood products;
− Any surgical procedure (management criteria).

We validated the near miss concept by testing the diagnostic accuracy (sensitivity, specificity, positive likelihood ratio, negative likelihood ratio, and odds ratio) using death as the reference variable.

### Statistical analysis

We calculated the frequency of maternal and neonatal near miss and used Fisher test to evaluate the association between the near miss cases and severe outcomes (all potentially life-threatening conditions and death). We calculated the maternal case fatality rate (number of maternal deaths divided by the number of deaths plus the number of maternal near miss) and neonatal case fatality rate (the number of neonatal deaths divided by the number of deaths plus the neonatal near miss number). Subsequently, we performed an ordinal and binomial logistic regression, with the independent variables comprising those that had *P* < 0.1 in the univariate analyses. We used the neonatal outcomes with three categories (no complications vs near miss vs death), as the dependent variable for the ordinal regression. For the binomial logistic regression, we combined near miss and death and used two categories (no complications vs near miss plus death), considering *P* < 0.05 as statistically significant. We used the WHO criteria to classify maternal morbidity and near miss [11].

For the binary logistic regression, we used the previously defined independent variables, including maternal characteristics (e.g. age, past conditions) and grouped maternal pre-gestational pathologies (due to the very low individual prevalence of each of diseases) (see Table S1 in the **Online Supplementary Document** for distribution of each condition). All parameters were estimated in relation to the reference category as the one with the best outcome potential (e.g. for education, the reference group included women attained the highest schooling possible; for gestational age, women between 37 and 40 weeks; for age, women 24–29 years of age).

We constructed a receiver operating characteristics (ROC) curve to evaluate the prediction capacity of the binomial logistic regression model by visualising the sensitivity (S) and specificity (E) of the logits. A true positive (PV) would be considered when the model predicted a woman would experience a neonatal mortality/near miss, and this in fact occurred, while a false positive (FP) would be the one which was predicted to experience a neonatal mortality/near miss but did not. A false negative (FN) would occur when a mother did not present any of the predictive characteristics chosen, but the baby died, while a true negative would occur when the mother was not expected to experience a fetal mortality, and the fetus indeed survived. We calculated the area under the curve and its associated confidence intervals (CIs).

## RESULTS

**Figure 1:**
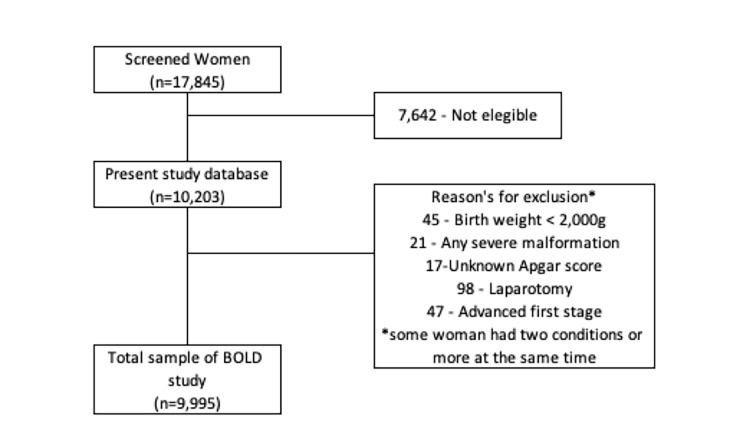
Flowchart for data sample.

The BOLD study initially collected data from 17 845 women screened between December 2014 and November 2015. In our sample, 7642 women were not eligible, leaving 10 203 for analysis (**Figure 1** and **Table 1**). We found that maternal and neonatal near miss data accounted for 0.1% and 10.1% of the study sample, respectively.

**Table 1:** Descriptive characteristics of women and their newborns

Women	n (%)
Country	
*Nigeria*	5063 (49.6)
*Uganda*	5140 (50.4)
Age in years	
*<20*	327 (3.2)
*20-24*	2373 (23.3)
*25-29*	3823 (37.5)
*30-34*	2546 (25.0)
*>34*	1130 (11.0)
*No information*	4
Marital status	
*Married*	9957 (97.6)
*Single/divorced*	243 (2.4)
*No information*	3
Number of Pregnancies	
*1-2*	5792 (57.0)
*>2*	4362 (43.0)
*No information*	49
Parity	
*0*	3533 (37.0)
*1-2*	4314 (45.0)
*>2*	1718 (18.0)
*No information*	638
Number of previous abortions	
*0*	6194 (71.5)
*1-2*	1599 (18.5)
*>2*	871 (10.0)
*No information*	1539
Number of previous cesarean sections	
*0*	8006 (93.5)
*1-2*	551 (6.4)
*>2*	9 (0.1)
*No information*	1637
Maternal outcomes	
*No complications*	7965 (78.1)
*Severe Morbidity*	2192 (21.5)
*Near miss*	11 (0.1)
*Death*	26 (0.3)
*No information*	9
Number of prenatal consultations	
*<8*	9626 (95.9)
*≥8*	412 (4.1)
Occupation status	
*Yes*	6792 (33.0)
*No*	3401 (66.0)
*No information*	
Education level*	
*1*	275 (2.7)
*2*	888 (8.8)
*3*	4323 (42.7)
*4*	4630 (45.8)
*No information*	87
**Newborns**	
Birth weight in grams	
*<2500*	345 (3.4)
*2500-3999*	9218 (90.5)
*≥4000*	624 (6.1)
*No information*	16
Newborn vital status	
*Alive*	10129 (99.3)
*Stillbirth*	72 (0.7)
*No information*	2
Apgar at 5° min	
*0-3*	68 (0.7)
*4-6*	214 (2.1)
*7-10*	9905 (97.2)
*No information*	16
Foetal presentation at delivery	
*Cephalic*	10061 (98.6)
*Breech*	112 (1.1)
*Transverse*	28 (0.3)
*No information*	2
Neonatal outcomes	
*Alive without complications*	9080 (89.2)
*Near miss*	1028 (10.1)
*Death*	72 (0.7)
*No information*	23

*1 – no education, pre-primary education, other; 2 – incomplete or complete primary education; 3 – incomplete or complete secondary education; 4 – incomplete or complete post-secondary/tertiary education or higher education.

We calculated the positive and negative likelihood, sensitivity, specificity, and odds ratios to compare diagnostic accuracy data using the chosen neonatal near miss data and found diagnostic performance to be similar between our sample using the BOLD database and two WHO cross-sectional studies: the Global Survey on Maternal and Perinatal Health (WHOGS) and the Multicountry Survey on Maternal and Newborn Health (WHOMCS) (**Table 2**). Meanwhile, the maternal and neonatal calculated case fatality rates were 70.2% and 6.5%, respectively (**Table 3**). Women who sustained increasingly more severe morbidities were at an increased risk of near miss or mortality outcomes (*P* < 0.05) (**Table 4**).

**Table 2:** Diagnosis accuracy for neonatal near miss classification*

	Present study		Pileggi-Castro et al. (2014)	
**Variables**	**Any pragmatic or management marker**	**Only pragmatic marker**	**Any pragmatic or management marker**	**Only pragmatic marker**
**Sensitivity (95% CI)**	99% (92–100)	93% (84–98)	92.8% (91.8–93.7)	79.1% (77.3–80.8)
**Specificity (95% CI)**	90 (89–90)	98% (97–98)	92.7% (92.6–92.8)	96.5% (96.4–96.5)
**Positive likelihood ratio (95% CI)**	9.69 (9.08–10.34)	40.9 (35.4–47.26)	12.7 (12.5–12.9)	22.3 (21.7–23.0)
**Negative likelihood ratio (95% CI)**	0.02 (0.00–0.11)	0.08 (0.03–0.17)	0.08 (0.07–0.09)	0.22 (0.20–0.24)
**OR (95% CI)**	600.6 (83.3–4330)	543.6 (216.6–1364.1)	163.4 (141.6–188.4)	103.1 (92.5–114.9)

OR – odds ratio, CI – confidence interval

*Cross tables at Table S2 available only in the **Online Supplementary Document**.

**Table 3:** Associations between maternal outcomes and neonatal near miss

	Neonatal outcomes				
**Women**	**No complications**	**Near miss**	**Total**	**OR (95% CI)**	*P*-value*
No complications	7411 (73.3)	495 (4.9)	7906	-	-
Morbidity	1639 (16.2)	523 (5.18)	2162	4.77 (4.17–5.46)	<0.001
Near miss	8 (0.08)	3 (0.03)	11	5.79 (1.2–20.56)	0.03
Death	15 (0.15)	7 (0.07)	22	7.06 (2.64–16.99)	<0.001
Total	9073	1028	10 101	-	-

OR – odds ratio, CI – confidence interval

*Fisher exact test.

**Table 4:** Associations between maternal outcomes and neonatal death.

	Neonatal death				
**Women**	**No**	**Yes**	**Total**	**OR (95% CI)**	***P*-value**
No complications	7906 (77.8)	41 (0.4)	7947	-	-
Morbidity	2162 (21.3)	28 (0.27)	2190	2.5 (1.52–4.04)	<0.0003
Near miss*	-	-	-	-	-
Death	22 (0.2)	3 (0.03)	25	27.3 (6.03–83.82)	<0.0003
Total	10 090	72	10 162	-	-

*No cases of maternal near misses occurring neonatal death.

We performed logistic ordinal regression using neonatal outcomes as the dependent and various maternal demographic and health characteristics as the independent variables (**Table 5**). We found that being at a gestational age of <37 or >41 weeks and having <8 antenatal consultations lead to a higher risk of adverse neonatal outcomes, while a maternal age of 30–34 years acted as a protective factor. We then performed binomial logistic regression using data form 8092 women by adding death with neonatal near miss as the dependent variable. In this model, the risk variables for unfavorable neonatal outcomes (near miss + death) were a gestational age of <37 or >41 weeks, having a low educational level, being overweight and obese, having a previous Cesarean section, having a previous abortion, and having a previous chronic condition. Meanwhile those who had one or more previous deliveries were at a reduced risk for adverse neonatal outcomes (Table S3 in the **Online Supplementary Document**).

**Table 5:** Ordinal logistic regression analysis for predicting neonatal outcomes (death, near miss, and no complications) based on maternal characteristics (n = 9821)*

Maternal characteristics	OR (95% CI)	*P*-value
Undernutrition	1.0 (0.83–1.24)	0.83
Overweight and obesity	1.12 (0.97–1.30)	0.10
Educational level 1	1.38 (0.92–2.01)	0.10
Educational level 2	0.87 (0.67–1.12)	0.31
Educational level 3	0.98 (0.85–1.13)	0.86
Gestational age <37 weeks	1.45 (1.13–1.85)	<0.01
Gestational age >41 weeks	2.04 (1.41–2.88)	<0.01
Age <20 y	1.11 (0.76–1.60)	0.55
Age 20–24 y	1.09 (0.92–1.29)	0.30
Age 30–34 y	0.77 (0.64–0.92)	<0.01
Age ≥35 y	1.00 (0.80–1.25)	0.98
Morbidity	4.60 (4.02–5.26)	<0.01
Maternal Near miss	3.73 (0.55–15.46)	0.10
Maternal mortality	10.93 (4.64–24.64)	<0.01
Occupation	1.02 (0.88–1.20)	0.75
Pre-natal consultations smaller than 8	1.90 (1.22–3.17)	0.01

CI – confidence interval, OR – odds ratio

*Educational level 1 – no education or pre-primary, other; Educational level 2 – incomplete or complete primary; Educational level 3 – incomplete or complete secondary education.

**Figure 2:**
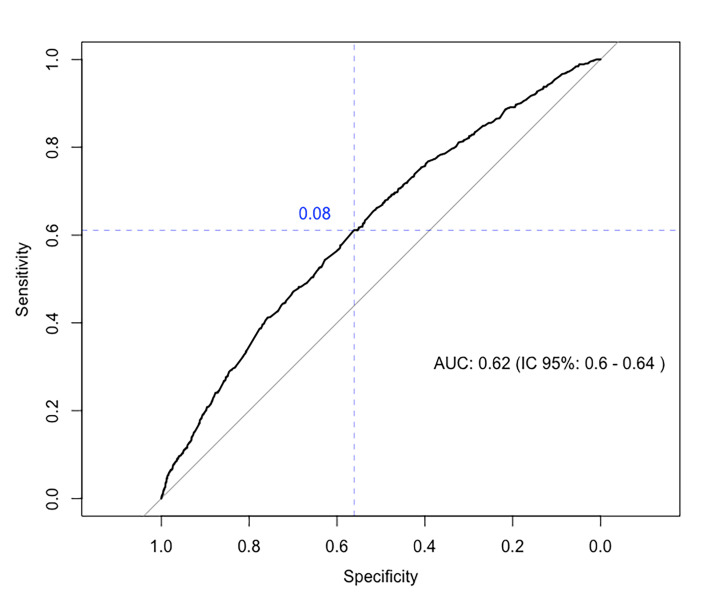
ROC curve of logistic regression.

We constructed an area under the curve (AUC) to better understand the behavior of the variables in the proposed model; it had an area of 0.62 (95% CI = 0.60–0.64) (**Figure 2**). It was observed that the variables are not able to adequately predict unfavorable neonatal outcomes once the values approach 0.5. This can be considered a low predictive performance.

## DISCUSSION

In this study, we validated the concept of a neonatal near miss and found a strong positive association between severe maternal and neonatal outcomes, meaning that more severe maternal clinical condition during labor correlated with greater the chances for the newborn sustaining a severe outcome.

### Near miss concept

The consolidation of the neonatal near miss concept by combining pragmatic and management indicators holds relevance for clinical setting. It can serve for health surveillance and care assessment, as a comparator between different facilities if routinely collected, and as an adjunct tool to evaluate severe cases of neonatal morbidity where neonatal death has becoming rare [12].

Given the novelty of the near miss concept and the absence of related studies, we analysed diagnostic accuracy by assessing sensitivity, specificity, positive and negative predictive values, as well as odds and likelihood ratios. First, we used sensitivity and specificity to accurately identify whether the newborn was classified as a near miss or not – tests with higher the sensitivity and specificity could more successfully confirm and rule out, respectively, the presence of a near miss [13]. We found both values to be consistent with this premise. Meanwhile, we observed a positive likelihood ratio close to 10 and a negative one <0.1, which represents convincing diagnostic evidence. Finally, odds ratios describe the relative chance of the event (death) among those who had the test positive to near miss when compared to the chance of the event in babies without the near miss outcome [14].

The concept of maternal near miss was developed to foster improvement in the health care of pregnant women around the time of delivery, and to identify and prevent maternal deaths [15]. Several studies have been conducted using this classification and critically analysed processes to improve the quality of care.

A study conducted in Paraná, Brazil analysed the near miss classification for women and identified the need for greater attention for pregnant women who were >35 years of age [16]. In Rwanda, applying near miss classification detected flaws in clinical practice and in the referral system [17]. In India, the use of the classification tool led to significant improvements related to mortality as it allowed for the detection of main system failures [18].

Using near miss in clinical practice can help advance understanding of the concept itself and allow for the implementation of life-saving interventions, as exemplified by a systematic review that demonstrated an improvement in maternal care in resource-poor settings through using a device that prevents shock in women who presented with postpartum haemorrhage [19]. Since one of the criteria of maternal near miss is blood transfusion, the use of this indicator might have allowed for finding an innovative solution to the problem.

### Prevalence of neonatal near miss

In a study conducted in Nigeria (one of the two countries encompassed by our analysis), Mbachu et al. [20] found that most women who delivered and died came from rural areas. They also did not seek assistance from skilled professionals due to their low educational status, suggesting that the maternal near miss problem in these regions could be resolved by improving the quality of care. This is supported by our findings, as the observed prevalence for near miss was small (0.1%) in more educated women (>45% with level 4).

In Nigeria, less than 40% of women had their newborns delivered using skilled labor professionals [21]. We found that women who delivered in a hospital setting had significantly reduced maternal mortality rates and near miss situations. It is therefore important to further investigate the reason why these women are unable to reach the required qualified health services and to determine whether they are sufficient to meet the demands of childbirth [22]. A successful example of this approach is demonstrated in Cambodia, where an increase in the number of ambulances led to improved transport of women to health services [23].

### Maternal and neonatal near miss

The relationship between maternal and neonatal outcomes has been explored previously. In 2013, Oliveira et al. [24] found a higher occurrence of fetal and neonatal deaths in patients with maternal near miss. Another prospective study conducted in Finland found a positive association between maternal near miss and stillbirths [25]. However, neither study explored the progressive association between maternal severity and the severity of the newborns.

In our ordinal regression model, we found that being 30–34-year-old served as a protective factor for neonatal near miss. This relationship can possibly be explained by the fact that most women in this range are well educated (88.6% with level 3 or 4 education). Likewise, education can be indirectly influenced by age, although this has not been explored by current research, to the best of our knowledge. Concerning education, numerous studies have already suggested that a higher maternal level of education is associated with a reduced risk of perinatal mortality [26-28]. Our findings thus indirectly support the hypothesis that having only a few years more of education led to improvements in undesirable outcomes in newborns.

We found that the risk factors for severe neonatal outcomes were gestational age <37 weeks or >41 weeks, maternal morbidity and mortality, and <8 prenatal consultations. It has been established that term delivery (between 37 and 41 weeks of gestation) reduces the risk of complications for the neonate. The WHO notes that preterm infants accounted for most deaths of children <5 years of age and were for approximately one million deaths in 2015 [29]. Several studies have shown that post-term births increase the risk of both neonatal and maternal morbidity and mortality [30-32], as was found in our analysis.

The WHO established that the threshold of <8 of antenatal visits reduces perinatal death by eight per 1000 births when compared to four visits [33]. Perhaps, this can be attributed to a greater opportunity for receiving antenatal care from skilled professionals thereby increasing the chances to receive counselling, obtain a risk assessment, and managing their conditions during pregnancy. However, the model chosen was not able to predict poor neonatal outcomes (**Figure 2**).

Notably, we found maternal mortality to be very high (70%), indicating that the number of women with maternal near miss was low and that those who presented were classified as more likely to die. By comparison, the neonatal mortality rate was 6%, suggesting a great discrepancy between health care for newborns and for women. This implies a need to better understand the environmental conditions present on order to achieve improvements in care, or to transfer mothers to units which provide care for childbirth [34]. However, given that the concept of near miss is quite broad, this might result in an over-estimation of near miss patients.

The regression models presented the following risk variables for a severe neonatal outcome: gestational age <37 weeks or >41, illiterate women, previous cesarean section, amniotic membrane status, fetal heart rate >120, uterine height <34 cm, and pathologies in the current gestation. The model also showed the protective effect of having one or more previous deliveries.

Kale et al. [35] found that that skin color (black), hemorrhage, hypertension, syphilis, lack of prenatal care, and cesarean section were associated with neonatal near miss and death. In our analysis, the risk factors for near miss and neonatal death were low education, gestational age <37 weeks or >41 weeks, overweight and obesity, having a previous cesarean section, and having some pathological conditions prior to gestation.

Cesarean section is an intervention that, according to the WHO, should be performed between 10% and 15% of deliveries in order to improve mortality rates [36]. A study of Scottish women with a previous cesarean section demonstrated that women with a previous cesarean section have an increased risk of uterine rupture [37] which may be one reason for serious neonatal outcomes in our sample.

In our study, the variable membrane status indicated that the membrane is prone to rupture. Meanwhile, most previous studies referred to premature rupture rather than to the status of the membrane itself. A study conducted in Brazil determined that premature rupture of the membrane contributes to premature births and, consequently, to respiratory and other complications [38]. However, it is not possible to know if these ruptures were premature or not, making it necessary to consider membrane status (whether or not it is rotated), since we found an association with severe neonatal outcomes.

### Study limitations

Our study has some limitations. The BOLD project was designed to respond to a specific maternal health need, and therefore collected little data regarding the newborns, which prevented further analyses. As it was conducted in hospitals, the severe outcomes are not representative of the study population, but of those who attend these units. Furthermore, the data were validated in two of the sites originally used for the near miss criteria, which may have generated a small bias. Likewise, there were no women with <34 weeks who gave birth, which reduced the scope of the neonatal near miss concept. However, we believe that the combined set was able to have the necessary diagnostic accuracy to test near miss classification, implying that our reduced gestational sample is not necessarily negative.

## CONCLUSIONS

The concept of neonatal near miss can be explored to compare the efficacy of care for neonates from different health care units. We also observed an association between maternal and neonatal severity which cannot be ignored. Both of these findings could be related to the quality of care provided. Maternal surveillance should be conducted to mitigate poor maternal and neonatal outcomes. This study stimulates implementation research to improve care and could support future global epidemiological studies related to the classification of nutritional status of mothers during delivery. This method alone can serve as an important tool for screening maternal and child health at childbirth.

## Additional material


Online Supplementary Document
