# Supplementary material for: Unveiling the strong positive relationship: Maternal characteristics and neonatal outcomes in the Better Outcomes in Labour Difficulty (BOLD) study – a secondary analysis validating neonatal near miss classification
Source: J Glob Health. 2024 Jan 19;14:04024. doi: 10.7189/jogh.14.04024 (PMC10802829; doi:10.7189/jogh.14.04024)
Supplement: Online Supplementary Document [file jogh-14-04024-s001.pdf]

Supplementary File S1

Number of women with each condition grouped as maternal previous pathologies

73 - Chronic hypertension
15 - Diabetes mellitus
442 - HIV +
10 - AIDS / HIV
6 - Chronic anaemia
34 - Obesity
5 - Heart disease
17 - Lung disease
2 - Kidney disease
32 - Other chronic conditions

Supplementary File S2

Cross tables

Cross table of Diagnosis Accuracy Test (Any pragmatic or management marker)

	Outcome (death) +	Outcome (death) –
Test +	68	1028
Test –	1	9080

*26 missing values (n=10,177)

Cross table of Diagnosis Accuracy Test (Only pragmatic marker)

	Outcome (death) +	Outcome (death) –
Test +	63	229
Test –	5	9880

*26 missing values (n=10,177)

Supplementary File 3

Binary logistic regression analysis for predicting neonatal outcomes (near miss + death and no complications) based on maternal characteristics

**Maternal Characteristics**	***Odds Ratio***	**CI**	**p-value**
Age <20 years	0.62	0.34 – 1.05	0.10
Age 20-24 years	0.94	0.75 – 1.18	0.60
Age 30-34 years	0.92	0.73 – 1.14	0.44
Age >35 years	1.23	0.91 – 1.65	0.17
Number of pregnancies >2	0.84	0.63 – 1.12	0.24
**Gestational Age <37 weeks**	**1.46**	**1.07 – 1.94**	**0.01**
**Gestational Age >41 weeks**	**2.26**	**1.55 – 3.20**	**<0.001**
**Educational Level 1**	**1.76**	**1.12 – 2.69**	**0.01**

Educational Level 2	1.04	0.74 – 1.43	0.83
Educational Level 3	1.08	0.89 – 1.30	0.43
Undernutrition	1.08	0.84 – 1.39	0.54
**Overweight and Obesity**	**1.23**	**1.02 – 1.47**	**0.03**
**Previous C-section =1**	**1.90**	**1.36 – 2.61**	**<0.001**
Previous C-section >2	2.70	0.14 – 18.90	0.38
**Previous abortion =1**	**1.25**	**1.00 – 1.56**	**0.05**
Previous abortion >2	1.11	0.78 – 1.56	0.56
**Number of births 1 or 2**	**0.56**	**0.44 – 0.70**	**<0.001**
**Number of births >2**	**0.55**	**0.37 – 0.80**	**<0.001**
**Previous conditions**	**1.83**	**1.37 – 2.41**	**<0.001**

Legend: n = 8092 women; Educational level 1 = no education or pre-primary, other; Educational level 2 = incomplete or complete primary; Educational level 3 = incomplete or complete secondary education. Previous condition (one or more of the following): chronic hypertension, diabetes mellitus, HIV, AIDS / HIV, chronic anaemia, obesity, heart disease, lung disease, kidney disease and other chronic diseases.
